# Telemedicine Public Reimbursement Models for National and Subnational Jurisdictions: Scoping Review

**DOI:** 10.2196/75478

**Published:** 2025-08-12

**Authors:** Evan Huang-Ku, Panchanok Muenkaew, Kinanti Khansa Chavarina, Yin May Tun, Zin Nwe Win, Wanrudee Isaranuwatchai, Saudamini Vishwanath Dabak, Natasha Howard

**Affiliations:** 1 Health Intervention and Technology Assessment Program Foundation, Ministry of Public Health, Department of Health, Building 6, 6th Floor, Mueang Nonthaburi, 11000, Thailand, 66 025904549; 2 Institute of Health Policy, Management, and Evaluation, University of Toronto, Toronto, ON, Canada; 3 Saw Swee Hock School of Public Health, National University of Singapore and National University Health System, Singapore, Singapore; 4 Department of Global Health & Development, London School of Hygiene & Tropical Medicine, London, United Kingdom

**Keywords:** telehealth, health financing, eHealth, mobile health, mHealth, telecare

## Abstract

**Background:**

Telemedicine has transformed health care delivery, offering improved access, efficiency, and potentially cost-effectiveness. However, wide-scale implementation is challenged due to multiple factors. Among these, reimbursements were reported to influence the scalability and sustainability of telemedicine.

**Objective:**

This study aimed to examine current payment models and reimbursement coverage for telemedicine in any national and subnational jurisdictions to inform the development of reimbursement policy.

**Methods:**

We conducted a scoping review using Arksey and O’Malley’s 6-stage method, including sources that discussed telemedicine payment methods reimbursed by public payers. To supplement the limited results, particularly from low- and middle-income countries in Asia, we conducted 5 stakeholder interviews with telemedicine providers or those with experience in telemedicine reimbursement models who added insights for India, Nepal, and Taiwan. Data were synthesized narratively.

**Results:**

We included 31 of 14,522 records screened. Most (n=22, 71%) records were published after 2020, were research studies (n=26, 84%), and discussed reimbursement in the United States (n=24, 77%). We categorized reimbursement coverage as the purpose of telemedicine, health conditions, patients’ nonhealth conditions, service providers, interaction participants, interaction modes, and technology used. Payment methods varied widely and included fee-for-service, capitation, bundled payment, and value-based models. Varying telemedicine reimbursement models adopted by countries reflect health service and care objectives along with health system characteristics. Payment mechanisms were linked to telemedicine services or broader health care delivery, with each presenting unique advantages.

**Conclusions:**

Workable telemedicine reimbursement is a critical enabling factor in expanding health care access by incentivizing provider participation, ensuring financial sustainability, promoting equity in access, and aligning telemedicine with broader health goals. This review provides a starting point for countries in designing a telemedicine reimbursement model specific to population needs and health system capacity. Policy makers are encouraged to leverage these insights in adapting telemedicine reimbursement to their context.

## Introduction

Telemedicine has revolutionized health care delivery by leveraging information and communication technology to enhance access, improve efficiency, and support the management of chronic diseases [1]. It has also demonstrated potential cost savings by reducing travel, hospitalizations, and unnecessary in-person visits [1]. The COVID-19 pandemic highlighted the benefits of telemedicine, which played a critical role in maintaining access to care and accelerating its adoption globally [2-4]. Despite its promise, there are numerous challenges in implementing telemedicine. These include inconsistent regulations, lack of governance, infrastructure limitations, workforce shortages, and financial constraints [3,5]. Among these barriers, reimbursement issues were reported to influence provider and patient participation, as well as the scalability and long-term sustainability of telemedicine programs [5-7].

While there is a growing body of literature on telemedicine reimbursement, it focuses on specific regions or countries and targets particular diseases or conditions [8-10]. Furthermore, a recent scoping review of the topic lacked sufficient detail to comprehensively describe the extent, nature, distribution, and key elements of telemedicine reimbursement [11]. This gap limits the ability of health care payers and policy makers to understand the elements of telemedicine reimbursement models across diverse contexts.

As a part of the World Health Organization’s Country Cooperation Strategy program on Digital Health in Thailand, aiming to support the development of digital health and health information systems, the research team conducted this study to inform the National Health Security Office (NHSO)—the administrator of Thailand’s largest public health insurance scheme—of potential means to improve reimbursement for telemedicine in Thailand. A consultation meeting with the NHSO identified their need for a foundational understanding of global telemedicine reimbursement models. This study, therefore, aimed to review and summarize current telemedicine payment models and reimbursement coverage to refine the design of national telemedicine reimbursement.

## Methods

### Overview

We conducted a multimethod study consisting of scoping literature review—following Arksey and O’Malley’s six-stage method [12], as described in Durrance-Bagale et al [13]—and stakeholder interviews to inform and help fill gaps in literature findings, particularly for low- and middle-income countries (LMICs) in the Asia region.

### Scoping Review

#### Defining the Research Question

We conducted a consultation meeting identifying the following components needed by NHSO: payment method, rationing criteria (ie, eligibility criteria), type of technology or services reimbursed, monitoring and evaluation methods, and fraud prevention measures. We developed a research question to address the breadth and varied components of telemedicine reimbursement models and policy-making requirements: “What are the scope and main findings of existing literature on reimbursement models, benefits, and limitations for telemedicine services used in national and subnational jurisdictions?” Table 1 presents definitions we used to guide the research. Multimedia Appendix 1 shows telemedicine reimbursement components.

**Table 1.** Study definitions.

Terms	Definitions
Telemedicine	Telemedicine is a branch of telehealth that focuses solely on clinical health care delivery [14]. It uses information and communication technology to facilitate interactions between patients and health care providers, or among health care providers acting on behalf of the patient for purposes such as diagnosis, treatment, and follow-ups. In contrast, nonclinical health care includes data management, health education, and public health functions that do not involve direct patient care. These interactions support services related to health promotion, disease prevention, diagnosis, disease monitoring, and effective patient rehabilitation. Telemedicine encompasses three primary forms: Store and forward (asynchronous telemedicine): operates similarly to email, where patient health records are shared with physicians in a secure and efficient manner [15]. Remote monitoring: enables patient monitoring through technological devices, primarily used to manage chronic diseases such as diabetes, asthma, and cardiovascular diseases [15]. Real-time interactive services allow patients to access consultations with doctors through one-on-one videoconferencing services [15].
Payment method	Payment methods describe how providers are paid for their services, which can be broadly classified as prospective (eg, capitation) or retrospective (eg, fee-for-service) [16].
Reimbursement pathway	This is a structured process that health care providers must follow to obtain payment for their services. This pathway outlines the steps from the initial submission of a claim to the final receipt of payment, including necessary documentation, approval processes, and any necessary appeals if a claim is denied.
Jurisdiction	The authority of a court or official organization to make decisions and judgments [17].

#### Identifying Relevant Sources

We searched 6 databases systematically (ie, PubMed, EMBASE (Ovid), Web of Science, Scopus, EBSCO Global Health, and ECONLIT), using search terms and syntax for telemedicine and reimbursement, adapted to the subject headings and terminology for each database. The search strategy for all 6 databases can be found in Multimedia Appendix 2. It was developed in consultation with a London School of Hygiene and Tropical Medicine librarian to improve specificity. In total, 10 studies from the final search strategy iteration in PubMed were reviewed independently by WI to confirm search strategy breadth and comprehensiveness.

#### Sources Selection

We uploaded search outputs to the Covidence (Veritas Health Information Ltd) literature review software for deduplication and screening. Table 2 shows our eligibility criteria. To increase consistency among researchers, titles or abstracts were independently double-screened by any two researchers among EH-K, PM, YMT, ZNW, KKC, and SVD, until more than 80% agreement in screening decision was achieved. Conflicts were resolved through team discussions until consensus was reached. Full texts were screened using the same approach.

**Table 2.** Eligibility criteria.

Criteria	Inclusion criteria	Exclusion criteria
Concept	Payment method of telemedicine services	Other reimbursement components of telemedicine services without any information on the payment method
Context or settings	National and subnational levels Telemedicine technology and services reimbursed by a public (government) payer	Supranational level Nontelemedicine technology and services reimbursed by public (government) or private (nongovernment) payer Telemedicine technology and services reimbursed by private (nongovernment) payers
Population	Individuals accessing any type of telemedicine service for any health condition	Individuals using services not facilitated by information and communication technology Individuals using information and communication technology–facilitated services not related to health Telemedicine used for nonhumans
Source type	Peer-reviewed studies Conference abstracts Books Reports Policy documents Guidelines and dissertations or theses.	Literature reviews and opinion sources (ie, news studies, editorial or opinion pieces, and encyclopedia entries)
Time period	All	N/A^a^
Language	No restriction	N/A

a Not applicable.

#### Charting Data

The 6 researchers (EH-K, PM, KKC, YMT, ZNW, and SVD) used Excel (Microsoft Corp) to extract source identifiers, relevant characteristics (eg, publication year, source type, and jurisdiction), and findings. Findings were extracted iteratively into reimbursement components as described in the Multimedia Appendix 1.

To pilot data charting, each of the 6 researchers extracted data from 2 of the 12 randomly selected papers, followed by a thorough discussion to reach consensus. Once data charting began, EH-K checked the first 5 sources charted by each researcher to improve data extraction quality and consistency.

### Stakeholder Interviews

#### Interview Guide Development

We developed an interview guide based on the research question and reimbursement components from the NHSO consultation (Multimedia Appendix 1). To refine questions, we piloted the guide with one of the authors (EH-K), who has experience providing telemedicine services.

#### Sampling and Recruitment

We used purposive and volunteer sampling by first emailing a seed list of 14 stakeholders identified from the Health Intervention and Technology Assessment Program Foundation (HITAP) network, which largely consists of Asian LMICs. To recruit additional stakeholders, we posted an invitation and study description on HITAP’s social media pages, calling for individuals with relevant experience or insights. We defined eligible candidates as telemedicine implementers (ie, implementing telemedicine in their scope of work) or providers with experience or information on telemedicine reimbursement. We received five expressions of interest, for which all candidates were deemed to be eligible. Profiles of the informants are available in Multimedia Appendix 3.

#### Data Collection

Interviews were conducted in English from August 6 to 23, 2024, over Zoom (Zoom Video Communications Inc). They lasted 45‐60 minutes, were recorded using Zoom, and transcribed using Otter.ai (Otter.ai, Inc) after anonymization. Stakeholders were asked to share links to websites or documents they considered relevant for the research team review (eg, government websites and unpublished documents). Written responses were collected from one stakeholder whom we were not able to interview. Transcripts were cleaned and validated by YMT and ZNW.

#### Collating, Synthesizing, and Reporting

We reported review screening using the PRISMA-ScR (Preferred Reporting Items of Systematic Reviews and Meta-Analyses Extension for Scoping Reviews) [18,19]. We summarized the extent, nature, and distribution of eligible sources descriptively. We first synthesized literature findings data, and then interviewed transcripts narratively, using a qualitative descriptive approach guided by the research question and data extraction categories (Multimedia Appendix 1). We reported relevant excerpts and insights from literature and stakeholders in the same categories. To improve internal validity and credibility, we triangulated data from interviews against literature, documents, and websites [20]. All stakeholders provided information for jurisdictions not identified from the review, except for the United States and Canada.

### Ethical Considerations

We obtained written informed consent from all stakeholders prior to the interview, after sharing the study information sheet and discussing any questions. Interviewees’ personal details were anonymized for analysis. Interview data were stored in a secure web-based repository managed by the HITAP and were accessible only to the research team. Ethics approval was provided by the Institute for the Development of Human Research Protection in Thailand (IHRP no. 041‐2567, COA no. IHRP2024033).

## Results

### Source and Participant Characteristics

**Figure 1.**
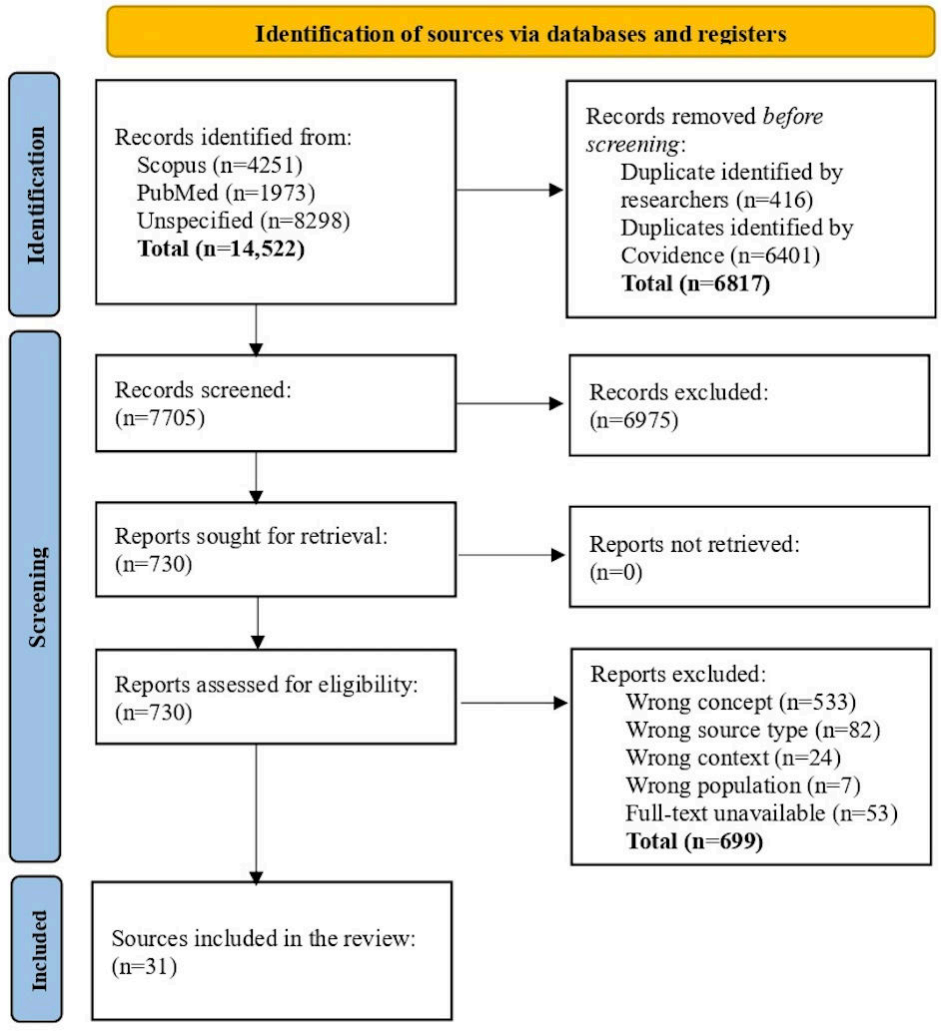
Records flowchart.

A total of 31 eligible sources are included from 14,522 identified (Figure 1). Most (n=22, 71%) records were published after 2019. Research studies were the most common source type (n=26, 84%), and conference abstracts, books, policy documents, and research letters constituted the rest of the source types. The United States (n=24, 77%) was the most reported jurisdiction. Two sources reported on more than one jurisdiction. Table 3 shows source characteristics.

**Table 3.** Characteristics of reviewed papers

Characteristics	Frequency (n=31), n (%)
Year published	
≤2019	9 (29)
2020	5 (16)
2021	4 (13)
2022	3 (10)
2023	5 (16)
2024	5 (16)
Source type	
Book	1 (3)
Conference abstract	2 (6)
Research study	26 (84)
Other^a^	2 (6)
Jurisdiction	
Australia	1 (3)
Belgium	1 (3)
Canada	1 (3)
Sweden	1 (3)
Switzerland	1 (3)
United Kingdom	1 (3)
United States	24 (77)
More than one jurisdiction^b^	1 (3)

a Includes policy documents and research letters.

b Includes Denmark, Estonia, Germany, France, Italy, the Netherlands, Spain, and the United Kingdom.

Five stakeholders participated, four via interview—with one providing additional information via email—and one via email only. Stakeholders represented diverse professional roles and geographical regions: telemedicine implementer from India (n=1), regional advisor for digital health in the United States and India (n=1), health care provider from Nepal (n=1), researcher from Taiwan (n=1), and health care facility manager from Canada (n=1). Multimedia Appendix 3 shows participant characteristics.

### Context and Scope of Reimbursement Coverage

#### Insurance

In the United States, Medicare and Medicaid were the reported public insurance programs, with Medicare administered at the federal level and Medicaid at the state level. In other jurisdictions, we found telemedicine publicly reimbursed at the federal level in Germany, Australia, Sweden, Belgium, the United Kingdom, and Switzerland, and at the provincial level in Ontario, Canada. As per stakeholders, the National Health Insurance (NHI) covered telemedicine in Taiwan, and publicly funded telemedicine was available in India and Nepal. In India, the central government funded the capital expenses for the national telemedicine (ie, Sanjeevani) while state governments managed the operational costs. Similarly, in Nepal, telemedicine services were supported through collaborative efforts between national and local governments to expand health care access in remote and underserved regions.

This review found multiple coverage aspects for telemedicine reimbursement that we categorized into telemedicine purposes, health conditions, patients’ nonhealth condition, service provider, participant interactions, interaction modes, and technology, which are described below.

#### Telemedicine Purposes and Health Conditions

Our reviews and interviews reported that reimbursable telemedicine services are often for diagnosis, treatment, follow-up, patient support, and management, indicating telemedicine’s critical role in direct clinical care [21-43]. Practices for these purposes were often associated with chronic conditions to provide ongoing care and prevention of disease progression, providing accessible care for patients with long-term health needs [21,22,24-26,28,29,33-35,42,43]. For example, the United States reimbursed remote care and referral coordination, medication management by phone call or secure SMS text message, self-care plan management, and other patient-provider communications through phone, text, and patient portals under the chronic care management program [22,33]. Telemedicine for prevention and promotion was reported to be reimbursed for patient self-care management [22] and school health–related programs [28]. Reimbursable telemedicine services for rural emergency departments in the United States also include the arrangement of interhospital transfer when necessary for patients with sepsis [32].

#### Patient’s Nonhealth Condition

From this review, 10 papers specified patients’ nonhealth eligibility criteria for reimbursement [24,32,34,38,41,42,44-47]. Within these papers, residing in remote or underserved areas as a patient was one of the commonly mentioned eligibility criteria (n=3, 27%) in the United States [38,42,45]. However, the United States has modified its regulations to broaden the geographical scope for telemedicine reimbursement. The Bipartisan Budget Act of 2018 expanded Medicare coverage, allowing patients in urban areas to also access treatment for acute stroke [42]. Medicare also had strict requirements on the types of facilities for which the patients could receive telemedicine, which was that the patients had to be in providers’ offices, hospitals, critical access hospitals, rural health clinics, federally qualified health centers, hospital-based or critical access hospital-based renal dialysis centers, skilled nursing facilities, and community mental health centers when meeting with the distant providers [42].

Finally, eligibility could also extend to the patient’s status as new or existing to the health provider. In Taiwan and India, a prior relationship must have been established between the provider and the patient for subsequent follow-ups to be conducted using telemedicine. In other cases, telemedicine was only available to existing patients receiving care in the context of schizophrenia and related psychotic disorders [24].

#### Service Provider

A total of 23 (81%) sources reported the health workers who delivered reimbursable telemedicine services [22-37,39,40,42,43,46,48,49]. Only certain health workers were mentioned (one source mentioned more than one health worker), with physicians being the most frequent type that delivered reimbursed services (n=17, 74%) among these sources.

#### Participant Interactions

All jurisdictions from the literature and interviews reimbursed telemedicine when the interaction was between providers and patients. In the United States, Canada, and India, telemedicine between providers, such as consultations, referrals, and getting second opinions, was also reimbursed [37,42]. In Canada, provider-to-provider interaction was done through a secure messaging server where primary care doctors or nurses could request opinions from specialists [37]. In the United States, provider-to-provider consultation occurred from a hub to a spoke in remote regions. For example, an experienced emergency physician or nurse provides on-demand consultation to emergency departments in remote hospitals, particularly for patients with uncommon or severe conditions [32]. In India, this interaction meant that providers at the spokes (ie, state-level health facilities) provided assisted teleconsultation to the patients connected to the specialists at the hubs (ie, secondary or tertiary-level health facilities).

#### Interaction Modes

A total of 21 (68%) sources described the temporal modality reimbursed for telemedicine, with one source describing more than one modality [22-28,30-32,35,37,38,40-45,47,49]. Most of the interactions took place in real-time (n=19, 90%), using audio, video, or other equipment, much like a face-to-face visit, followed by store-and-forward, where information (ie, transmitting a digital image from one provider to another) was used later for diagnosis or treatment (n=6, 28%), and remote monitoring (n=6, 28%) where the provider had ongoing access to patients’ data to monitor trends or facilitate interventions. Medicare defined telemedicine as real-time interactive audio-visual communication between the provider and the patient, which meant store-and-forward and audio-only telemedicine would not be eligible for reimbursement [42].

#### Technology

A total of 19 (61%) sources described the technology in reimbursable telemedicine service, with more than one technology described in one source [22,26,27,29-33,35,37,38,40,42-44,48,50,51]. The most frequent technologies used were videoconferencing (n=15, 79%) followed by audio-only (ie, phone calls; n=14, 74%), software apps (n=3, 16%), messaging services (n=3, 16%), website (n=1, 5%), and the physical device (ie, implant device and insulin pumps; n=1, 5%). According to an interviewee, Taiwan allowed the use of commercial videoconferencing platforms and mobile apps, while India only provided free telemedicine services through eSanjeevani. In the province of Ontario, Canada, the Ontario Telehealth Network used a website-based platform to connect providers with patients from their home or community-based settings to conduct clinical visits (Table 4 and Multimedia Appendix 4).

**Table 4.** Telemedicine reimbursement coverage aspects^a^.

Coverage aspects	Sources by jurisdictions mentioning the criteria			
	United States	Canada	Europe	Asia and Australia
Purpose				
Prevention or promotion	✓			
Diagnosis, treatment, follow-up, patient support, and management	✓✓	✓✓	✓	✓✓
Administrative services	✓			
Health condition				
Physical health	✓✓	✓✓	✓	✓✓
Mental health	✓	✓✓	✓	✓✓
Patient’s nonhealth condition				
Residence (rural or urban)	✓	✓		✓
Established relationship with care provider^b^	✓			
Vulnerability	✓		✓	
Service-related^c^	✓			
Service provider				
Physicians	✓✓	✓✓	✓	✓✓
Nurses	✓✓	✓✓	✓	✓
Pharmacist	✓		✓	
Social worker	✓		✓	
Other^d^	✓		✓	
Participant interactions				
Provider to patients	✓✓	✓✓	✓	✓✓
Provider to provider	✓✓	✓		✓✓
Interaction mode				
Interactive	✓	✓	✓	✓✓
Store and forward	✓	✓		✓
Remote monitoring	✓		✓	
Technology				
Video-based	✓✓	✓	✓	✓✓
Audio-based	✓✓	✓	✓	✓✓
Software apps	✓		✓	✓
Messaging services	✓		✓	
Physical devices			✓	
Website	✓	✓		✓

a The table includes only papers mentioning the criteria, and one paper may mention more than one criterion. A single check (✓) indicates the source is from a scoping review or stakeholder interview. Two checks (✓✓) mean it comes from both methods. The references used to generate this table are available in Multimedia Appendix 4.

b An established relationship refers to a patient who is already registered with the provider.

c The service should not originate from a related service that was provided in the previous 7 days nor leading to a service in the next 24 hours.

d This includes coaches, care managers, registered dietitians, physician assistants, speech and language therapy, and other health professionals (no specific profession mentioned).

### Payment Components

#### Payment Method

The payment method is classified based on the unit of reimbursement (eg, payment per service item or fee-for-service [FFS]) [52], except for two sources that reported prospective payment systems (ie, based on relation to providers’ income and cost for providing service) [52] without providing more details [35,47]. A summary of payment methods, the jurisdictions that used these methods, the health conditions they addressed, their pros and cons, and the technologies eligible for reimbursement under each method is provided in Table 5 and Multimedia Appendix 5.

**Table 5.** Reported telemedicine payment methods^a^.

Payment methods	Conditions or services	Pros	Cons
Prospective payment	Chronic conditions	Not reported	Not reported
Fee-for-service and payment per contact	Chronic conditions, dementia, mental health, COVID-19, emergency sepsis	Relatively preferred by service providers as it incentivizes service volume	Prioritizes volume over quality, which may trigger overuse of services
Capitation and episode-based	End-stage renal disease-related services	Maximizes the number of patients and may reduce the costs at the margin, as providers are expected to manage the episode within the prospective payment reimbursement formula	Not reported
Case-based and activity-based funding	Inpatient services, cancer services	Not reported	Not reported
Blended method^b^	Primary care services	Not reported	Not reported
Value-based payment			
Accountable Care Organization	Chronic care management, psychiatric and addiction, postdischarge monitoring, immunization, and minor procedures	Prevent unnecessary hospitalizations and adoption of low-value technology	May discourage initial adoption of new technologies due to cost concerns, and providers may face financial risk since they will be responsible for managing care within a fixed budget
Quality-adjusted outcome capitation	Primary care services	Offers the benefits of both outcome-based payment and capitation	Not reported
Bundled payments	Chronic condition and intensive critical care	Not reported	Not reported
Unspecified value-based method^c^	Elective care, emergencies, outpatients, and chronic conditions	Improve health outcomes (ie, amount of payment is linked to predefined health outcomes) and reduce health care resources (ie, resources will be allocated to technologies or services that only produce predefined health outcomes)	Not reported

a The references used to generate this table are available in Multimedia Appendix 5.

b Blended method refers to a mix of payment methods (eg, fee-for-service+capitation).

c Sources that reported value-based model but did not clarify the type.

The most frequently reported method was the FFS model (n=19, 59%) used in the United States, Canada, Belgium, Estonia, Germany, the Netherlands, and Switzerland [22-24,29-34,36-38,41,43,45,46,48,50,51]. The FFS model refers to paying providers for each service delivered, such as consultation, tests, or procedures [50]. A similar model, payment per contact, was used in Sweden to incentivize the service volume [44]. The FFS payment method was said to encourage the roll-out of telemedicine because providers were incentivized to deliver more telemedicine, as per the stakeholders in Nepal and Taiwan.

*The government realizes that not providing incentives (fee-for-service) to specialists would prevent the country from adopting telemedicine*.

[Informant from Nepal]

*The FFS model incentivizes health care providers to offer telemedicine services as they are reimbursed for each service provided. This can encourage the adoption and expansion of telemedicine services, particularly in areas where access to health care is limited*.

[Informant from Taiwan]

A stakeholder, along with our review, reported the downside of FFS, noting that it could encourage the unnecessary overutilization of telemedicine. This overutilization could result in a financial burden for the payer without providing additional health benefits for patients [22,30].

*The FFS model may create an incentive for health care providers to overutilize telemedicine services to maximize their reimbursement. This could lead to unnecessary consultations or procedures, potentially increasing health care costs and burdening the system*.

[Informant from Taiwan]

The capitation method was reported in 2 (6%) sources [29,38], where providers receive per capita payment for the registered population during a specific period for a fixed reimbursement amount [29]. A similar model to capitation, payment per episode (ie, occurrence of symptoms of an illness) model, is reported to be used in the United States by home health agencies’ telemedicine services [49]. Providers need to manage patients’ episodes within the reimbursement formula and can increase their reimbursement amount with more patients [49].

The case-based method was reported to be used in some European countries for inpatient services [34]. The diagnostic-related groups system was reported to be used in these countries to classify the diagnoses [34]. A similar model to case-based is activity-based funding that was reported to be used in Australia [26] and Denmark [34]. In this model, providers are paid for hospital activities (eg, phone calls by pharmacists).

The blended method, where payers use more than one payment method for a service, was reported in the United States [40] and some European countries [34]. In the United States, the providers of the Comprehensive Primary Care Plus program received payment through FFS, a monthly prospective risk-adjusted per-beneficiary care management fee, and an annual prospective performance-based incentive payment to account for nontraditional visits such as telemedicine [40]. In Denmark, Estonia, Germany, and the Netherlands, primary care services received payment through a combination of FFS and capitation, while a combination of capitation, cost reduction, and outcomes-based incentives was applied for primary care services in Italy [34].

Another payment method is value-based, which refers to a payment method that ties the amount paid to the provider to the results they deliver. The review reported several types of value-based payment models: Accountable Care Organization (ACO) [21,28,43,48,50], quality-adjusted outcome capitation [34,50], and bundled payments [21,27,34,42,48]. Although three sources did not identify the type [25,34,39]. Four sources described, broadly, the use of value-based payment for telemedicine, which is said to reduce unnecessary treatments, promote the efficient use of resources, and lead to long-term cost savings [21,25,43,48].

The ACO method has only been applied in the US setting. In an ACO model, providers are accountable for costs and outcomes for their clinical decision-making [48]. Rambur et al [48] described three categories of ACO: risk-sharing ACOs, full risk-bearing ACOs, and upside-only ACOs. In the risk-sharing scheme, payers and providers benefit from savings on cost containment, receive extra payments if their spending is below a set benchmark, and also share any financial losses [50]. In the full risk-bearing scheme, providers have to manage their care delivery themselves, and revenue to the providers may be in a fixed global budget or a collective cap for a population or service [48]. In the upside-only scheme, providers benefit from savings by managing costs and are not liable for any costs that exceed projections [48].

Bundled payments are used in the United States [21,27,34,42,48] and the Netherlands [34]. Providers receive a lump sum payment to cover all services involved in an episode of patient care [34,48]. One advantage of this payment method was promoting coordinated care and linking reimbursement to quality and cost reduction outcomes, exemplified in the case of tele-intensive care unit, where a monthly bundled payment from Medicare and Medicaid covered the appropriate mix of critical care specialists, subspecialty nurses, pharmacists, and technical support personnel [27].

#### Reimbursement Rate

Most sources described the rate as a numeric value in currency (n=7, 23%), followed by a comparison between the rate of telemedicine and in-person equivalent (n=7, 23%), and some other ways (n=2, 6%). An example of rates in numeric value was exemplified by the cost of an initial assessment conducted by a dermatologist and an ophthalmologist to be 44.45 and 45.85 Canadian dollars, according to the Ontario Schedule of Benefits dated 2021 [37]. Equal payment for telemedicine and in-person visits was described using the term “payment parity,” implemented in many US states and Taiwan, and was said to be able to encourage providers to offer telemedicine [51].

The remaining two papers described rates in some other ways: as components of the rate (professional fee, facility fee, and technical fee) or copayments required for telemedicine visits [42,44]. According to a stakeholder, basing telemedicine reimbursement on relative value units could result in lower payments compared to in-person visits. This was because relative value units primarily accounted for the time and effort involved, and telemedicine services were often perceived as requiring less of both when compared to in-person visits. This could discourage providers from offering telemedicine services, highlighting a potential barrier to its adoption.

#### Reimbursement Pathway

The reimbursement pathway refers to the process providers follow to obtain payment for their services, including steps from claim submissions to the final receipt of payment. This review found that the current procedural terminology codes were often required in the claims submission process to link the cost with services provided [22,23,31,33,38,41,42,45,46,50]. In Taiwan, the process began with the patient registering for the telemedicine service and verifying their eligibility for NHI coverage. Once eligibility is confirmed, the patient consults with a qualified health care provider who offers medical advice, diagnoses, and treatment recommendations. The health care provider then documented the service, including the diagnosis, treatment plan, and any prescriptions issued. Afterward, a claim is submitted to the NHI using standardized billing codes. The NHI reviews the claim for accuracy and completeness, and upon approval, disburses payment to the health care provider according to predetermined reimbursement rates.

#### Fraud Prevention

This review did not find any information pertaining to fraud prevention for telemedicine reimbursement. Our stakeholder from Taiwan mentioned that telemedicine followed the same fraud prevention strategies as other reimbursable services billed to the NHI Administration. These included strict eligibility verification, where health care providers must confirm patient eligibility before delivering services using a comprehensive database to confirm that only eligible individuals receive reimbursement. Claims could undergo both automated checks and manual audits to identify inconsistencies or unusual billing patterns. According to the stakeholder, NHI Administration also used advanced data analytics and artificial intelligence (AI) tools to detect fraud by analyzing billing data and identifying high-risk profiles of providers and patients. However, we could not identify any information in the public domain to corroborate the use of AI in this mechanism. Additionally, a whistleblower protection program encouraged individuals to report fraudulent activities while protecting those who come forward. Providers or patients found engaging in fraud faced penalties, including fines, suspension of NHI reimbursements, and even criminal prosecution.

### Technology Listing Process

**Figure 2.**
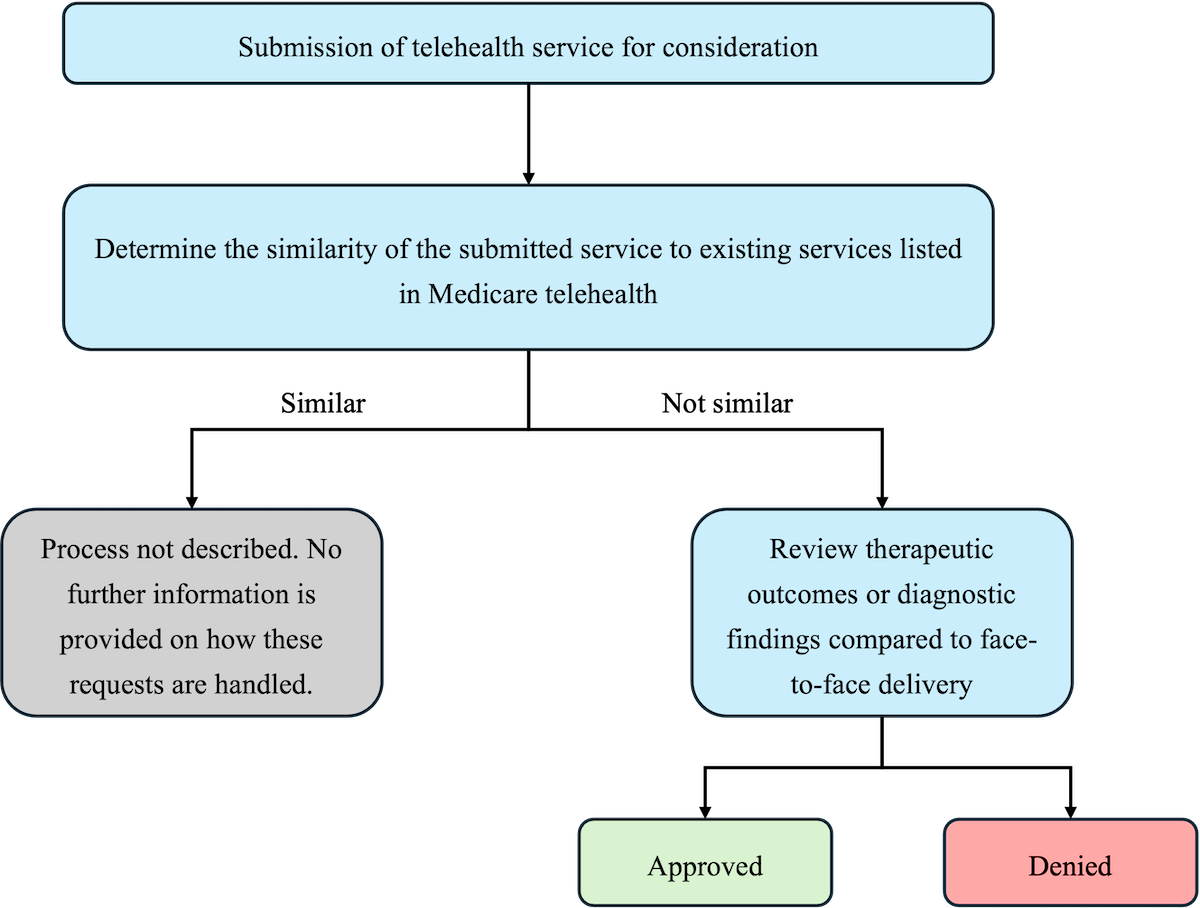
The process of adding services to the existing list of Medicare telehealth services. This figure was developed using data from one of the sources (adapted from Federal Register [38]).

One source described the listing process in the context of a health service as described in Figure 2 [38].

### Monitoring and Evaluation

We identified 10 (31%) sources that evaluated telemedicine services related to reimbursement [22,23,25,26,31,33,36,40,45,49], and 2 (5%) papers discussed the monitoring and evaluation policy under the Medicare program [38,43]. The outcomes reported by these sources include reimbursement rate [36,45,49], telemedicine uptake [23,25], health outcomes [31], resource use [26,31], claim challenges affecting patient experience [22], return on investment for chronic care management by pharmacists [33], and providers’ perception of whether reimbursement had a role in patient care improvement [40].

## Discussion

### Principal Findings

This study summarized the existing literature, supplemented by stakeholder insights, on public telemedicine reimbursement models, including the relevant components missing in a previous review [11]: scope of coverage, payment models, technology listing process, fraud prevention, and monitoring and evaluation of reimbursement models. Results from this study provide foundational information for policy makers and practitioners in Thailand to improve the design of national telemedicine reimbursement. Given the study design, we are able to outline policy considerations but are not in a position to evaluate the effectiveness of specific models [53-55]. Although this work was conducted for Thailand, we believe it has broader relevance for policy makers, researchers, and practitioners in other countries, given the global significance of telemedicine reimbursement.

The number of publications on telemedicine reimbursement remained steady before 2019 but experienced a sharp increase around 2020, presumably due to increased telemedicine implementation during the COVID-19 pandemic [56,57]. Most of the included publications focused on the United States, similar to the earlier scoping review on this topic [11]. We speculate that this study is dominated by US publications because of their economic viability and research capability to conduct such studies [58]. Conversely, the scarcity of research on telemedicine reimbursement in LMICs may be attributed to the limited adoption of telemedicine, explained by infrastructural barriers such as unreliable internet connectivity, lack of policies, unclear reimbursement rules, poor acceptability [59], research priorities in other areas, or nondocumentation of practices [60].

A notable strength of this study was the inclusion of stakeholder interviews to supplement literature review findings, which was particularly useful since there was very little literature on Asian countries and LMICs. Furthermore, we consulted public payer stakeholders to ensure that study results have policy relevance, which led to designing a well-suited data collection plan to identify elements of a reimbursement model for policy makers to consider. Below are the key elements identified that are applicable across different settings.

The first is equity in access, which aligns with the broader goal of addressing geographic disparities—and is consistently emphasized in telemedicine implementation globally. In the United States, telemedicine reimbursement policies often use patient remoteness or residence in underserved areas as an eligibility criterion. In India and Nepal, provider-to-provider telemedicine services are reimbursed to overcome barriers related to low digital literacy and inadequate infrastructure. For example, India’s eSanjeevani program enables patients to access telehealth services at local facilities, connecting them to specialists at secondary or tertiary level hospitals [61]. The design of telemedicine payment methods also plays a role in improving equity in access. FFS models may incentivize service provision and improve access but risk overtreatment and increased costs [22,44]. In contrast, capitation models may promote cost-efficiency but risk incentivizing undertreatment or the selective enrollment of healthier patients, potentially marginalizing more complex or vulnerable populations [52,62]. The US Comprehensive Primary Care Plus program introduced a monthly prospective, risk-adjusted care management fee paid per beneficiary to participating practices [40]. Although details on the risk adjustment methodology are limited in the available literature, this approach indicates an effort to account for variations in health care needs across different individuals and communities. Value-based payment models, despite their growing adoption, also pose equity concerns, as providers may preferentially serve patients with better expected outcomes [63], a risk that was not discussed in the included studies. Further exploration is needed to assess how effectively the method addresses disparities in health care utilization and outcomes within telemedicine systems.

The second element pertains to the quality of care and cost-effectiveness. Half the reviewed sources discussed value-based payment models that link provider compensation to patient outcomes, aiming to reduce unnecessary treatments and promote efficiency. These models, often tied to care programs using telemedicine, are applied in the United States and Europe. Emerging forms of telemedicine, such as remote patient monitoring and store-and-forward systems, have also been reimbursed. These models not only offer distinct capabilities, such as continuous data collection and asynchronous consultation, but may also enhance patient outcomes. Public payers should consider these innovations, as they hold the potential for improving health care delivery and efficiency while addressing diverse patient needs.

The third element is the rate of reimbursement. Many sources present reimbursement rates in monetary terms, which can be challenging to interpret across different countries due to variations in economic status, health care costs, and living standards. However, many sources emphasize that telemedicine reimbursement should be at the same rate as in-person services. This is to prevent bias toward a specific modality and promote telemedicine integration into standard health care practices. This reflects the ongoing debate over whether telemedicine or in-person visits yield better health outcomes and utilization [64,65], with some noting that telemedicine is not suitable for certain procedures [66,67].

Other elements identified in this study were patient convenience, comfort, and privacy when determining the health conditions eligible for telemedicine reimbursement. For example, telemedicine for chronic disease management that usually involves maintenance check-ups is typically reimbursed in most of the countries in this study. Mental health consultations—where in-person visits may pose ethical or privacy concerns—have been included in telemedicine reimbursement in some settings, reflecting efforts to address these challenges [68].

Policy makers in Thailand were interested in learning how capital costs, that is, investment requirements by health facilities to implement telemedicine, including the acquisition of equipment, technology, and spaces to conduct telemedicine. Although no such information was found in this review, some insights were shared by stakeholders. In the United States, large academic centers may receive grants from the Department of Health and Human Services, and in India, the capital cost to develop the national-level telemedicine platform eSanjeevani was fully borne by the Ministry of Health and Family Welfare. Their insights suggest that government support was involved in the adoption of telemedicine in these countries.

### Limitations

Several study limitations should be considered. First, we did not expand our search for gray literature outside of the databases and excluded reviews due to resource constraints, which may limit the comprehensiveness of the study. Second, we did not find relevant literature on fraud prevention measures. However, as explained by a stakeholder, this could be expected since publishing such data may pose a significant security risk to public payers. Third, most studies on telemedicine reimbursement are from high-income countries, especially the United States. While our stakeholder interviews included some Asian and LMIC experiences, their generalizability to Thailand remains limited due to differing health systems and reimbursement policies. Nonetheless, several principles remain relevant across contexts, such as using telemedicine to improve equity through rural access or adopting value-based payment to enhance care quality and efficiency. Given these limitations, we suggest countries use our findings as a starting point to identify relevant elements and conduct needs assessments to identify local priorities and constraints, and to develop context-specific reimbursement models. As we did not conduct a comparative study of payment methods, we recommend that future research assess how payment models can support telemedicine and broader health system goals in various settings, not limited to income status, funding mechanisms, levels of health care, and geographic remoteness.

### Conclusions

This study examined telemedicine reimbursement models across jurisdictions, although most findings were from the United States and other high-income countries. Key considerations in developing a reimbursement model include determining eligibility criteria, suitable health conditions, type of technologies, whether telemedicine is real-time, remote monitoring, or store-and-forward, and whether the interaction is between the patient and provider or between providers. Policy makers should note that payment parity may have a role in promoting telemedicine adoption. From a practical standpoint, countries should consider local needs to identify health priorities and resource constraints. The reimbursement elements identified in this review could be a reference point for designing a reimbursement model specific to population needs and health care system capacity. Future research could assess the comparative advantages of the various payment methods in differing settings to further inform reimbursement policy development and also evaluate the effectiveness of each model. The design of reimbursement of telemedicine is a critical enabling factor in expanding access to health by incentivizing provider participation, ensuring financial sustainability, promoting equity in access, and aligning telemedicine with broader health goals; policy makers are encouraged to leverage insights for their own context.

## Supplementary material

10.2196/75478 Multimedia Appendix 1 Components of telemedicine service and reimbursement.

10.2196/75478 Multimedia Appendix 2 Search strategy and keywords.

10.2196/75478 Multimedia Appendix 3 Informant profiles.

10.2196/75478 Multimedia Appendix 4 References for reimbursement coverage aspects.

10.2196/75478 Multimedia Appendix 5 References for payment methods.

10.2196/75478 Checklist 1 PRISMA-ScR (Preferred Reporting Items of Systematic Reviews and Meta-Analyses Extension for Scoping Reviews) checklist.

## Abbreviations

ACO: Accountable Care Organization
FFS: fee-for-service
HITAP: Health Intervention and Technology Assessment Program Foundation
LMIC: low- and middle-income country
NHI: National Health Insurance
NHSO: National Health Security Office
PRISMA-ScR: Preferred Reporting Items of Systematic Reviews and Meta-Analyses Extension for Scoping Reviews
