# Supplementary material for: Telemedicine Public Reimbursement Models for National and Subnational Jurisdictions: Scoping Review
Source: J Med Internet Res. 2025 Aug 12;27:e75478. doi: 10.2196/75478 (PMC12341443; doi:10.2196/75478)
Supplement: Multimedia Appendix 1 [file jmir-v27-e75478-s001.docx]

#

| Component | Definition |
| --- | --- |
| Reimbursement context and criteria | |
| Insurance | This component describes the public insurance scheme (e.g., Medicaid), alone of combined with a private insurance or out of pocket pain, in which country or geographic jurisdiction. |
| Purpose | This component details the purpose of using the telemedicine service, such as evaluation (diagnosing or treating), monitoring or follow-up or provider-to-provider consultation. |
| Non-health Eligibility | Criteria for reimbursable telemedicine unrelated to health conditions. |
| Health conditions | This component describes the indication (medical condition) covered by said reimbursement mechanism. I.e., in Thailand, 42 disease groups are reimbursable or medically related characteristics of the telemedicine service, such as speech and mental health therapy, preventive care, emergency care, medical specialty, support service. |
| Interaction | This component describes whether the interaction of telemedicine is provider-to-provider or provider-to-patient. |
| Service provider | This component describes the persons or systems that deliver health services using telehealth technology. |
| Time | The timing of telehealth services usage; for example, real-time or recorded. |
| Setting | The location where the telehealth services are put into use such as hospital, home, or community. |
| Technology | This component identifies the types of ICT used in telehealth services such as websites, mobile applications, only audio-based services, and video-based services. |
| Payment components | |
| Payment method | This component outlines how healthcare providers are paid for the service they deliver. These models include fee-for-service, capitation, bundled payments, pay for-performance (P4P), value-based care, and diagnosis-based payment (DRGs). |
| Reimbursement rate | This component captures the description of the reimbursement rate such as specific rate or comparison made with in-person fee. |
| Reimbursement pathway | This component describes the structured process that healthcare providers must follow to obtain payment for their services, such as the steps from the initial submission of a claim to the final receipt of payment, including necessary documentation, approval process, and any necessary appeals if a claim is denied. |
| Fraud prevention | These components describe any measure put along the reimbursement pathway to manage fraudulent conducts. |
| Service usage quota | This component explores considerations regarding the allowable number of telemedicine sessions per patient that are subject to reimbursement. |
| Pros & cons | This component identifies any explicit mentioning of benefit or limitation associated with a reimbursement mechanism. |
| Process for listing the technology in public insurances | |
| Listing process | This component describes the process or criteria required to list a technology in the public insurance. |
| Monitoring, evaluation, and recommendation | |
| Monitoring and evaluation | This component identifies explicit mentioning of any monitoring and evaluation of telemedicine reimbursement. |
| Recommendations | Recommendations to improving uptake of telemedicine services relevant to its reimbursement. |
