# Supplementary material for: Telemedicine Public Reimbursement Models for National and Subnational Jurisdictions: Scoping Review
Source: J Med Internet Res. 2025 Aug 12;27:e75478. doi: 10.2196/75478 (PMC12341443; doi:10.2196/75478)
Supplement: Multimedia Appendix 2 [file jmir-v27-e75478-s002.docx]

| Category | Keyword | Entry Terms |
| --- | --- | --- |
| Telemedicine | Telemedicine | Telemedicine; Tele-Referral; Tele Referral; Tele-Referrals; Virtual Medicine; Medicine, Virtual; eHealth; Telecare; Tele-Care; Tele Care; Tele-Intensive Care; Tele Intensive Care; Tele-ICU; Tele ICU; Mobile Health; Health, Mobile; mHealth. |
|  | Telehealth | - |
|  | Telemonitor | Remote monitoring |
|  | Teleconsultation | Teleconsultation; Teleconsultations; Consultation, Remote. |
|  | Telerehabilitation | Telerehabilitations; Virtual Rehabilitation; Rehabilitations, Virtual; Rehabilitation, Virtual; Virtual Rehabilitations; Tele-rehabilitation; Tele rehabilitation; Tele-rehabilitations; Remote Rehabilitation; Rehabilitation, Remote; Rehabilitations, Remote; Remote Rehabilitations. |
|  | Teletherapy | Teletherapy, Mental Health; Telepsychotherapy; Telepsychology; Counseling, Distance; E-Counseling; E Counseling; E-Therapy; E-Therapies; E Therapy. |
|  | Telediagnosis | - |
|  | Telepsychiatry | - |
|  | Telecomm | Telecom |
| Reimbursement | Reimbursement | Reimbursement; Incentive Reimbursements; Incentive Reimbursement; Pay for Performance; Performance, Pay for; Disproportionate Reimbursement; Disproportionate Reimbursements; Reimbursement, Disproportionate; Reimbursements, Disproportionate; Disproportionate Share Reimbursement; Disproportionate Share Reimbursements; Reimbursements, Disproportionate Share; Share Reimbursement, Disproportionate; Share Reimbursements, Disproportionate; Mechanism, Reimbursement; Mechanisms, Reimbursement; Reimbursement Mechanism; Reimbursement, Health Insurance; Third-Party Payments; Payment, Third-Party; Payments, Third-Party; Third Party Payments; Third-Party Payment; Health Insurance Reimbursement; Health Insurance Reimbursements; Insurance Reimbursement, Health; Insurance Reimbursements, Health; Reimbursements, Health Insurance; Third-Party Payers; Payer, Third-Party; Payers, Third-Party; Third Party Payers; Third-Party Payer; Reimbursement, Prospective; Prospective Reimbursement; Prospective Reimbursements; Reimbursements, Prospective; Fee-for-Service Reimbursement; Fee-for-Service Reimbursements; Reimbursement, Fee for Service; Reimbursements, Fee-for-Service. |
|  | Pay | Payments; Payment |
|  | Refund | - |
|  | Repay | Repayments; Repayment |
|  | Capitation |  |
|  | Bundled payments |  |
|  | Value-based care |  |
|  | Diagnosis-based payments |  |

**Search strategy: Medline via PubMed**

| Keyword | PubMed search syntax | No. of hits |
| --- | --- | --- |
| Telemedicine | 1. ((((((((((((((((Telemedicine[Title/Abstract]) OR (Tele-Referral[Title/Abstract])) OR (Tele Referral[Title/Abstract])) OR (Tele-Referrals[Title/Abstract])) OR (Virtual Medicine[Title/Abstract])) OR (Medicine, Virtual[Title/Abstract])) OR (eHealth[Title/Abstract])) OR (Telecare[Title/Abstract])) OR (Tele-Care[Title/Abstract])) OR (Tele Care[Title/Abstract])) OR (Tele-Intensive Care[Title/Abstract])) OR (Tele Intensive Care[Title/Abstract])) OR (Tele-ICU[Title/Abstract])) OR (Tele ICU[Title/Abstract])) OR (Mobile Health[Title/Abstract])) OR (Health, Mobile[Title/Abstract])) OR (mHealth[Title/Abstract]) | 51,023 |
|  | 1. (((((((((Telehealth[Title/Abstract]) OR (Telemonitor[Title/Abstract])) OR (Remote monitoring[Title/Abstract])) OR (Teleconsultation[Title/Abstract])) OR (Teleconsultations[Title/Abstract])) OR (Consultation, Remote[Title/Abstract])) OR (Telediagnosis[Title/Abstract])) OR (Telepsychiatry[Title/Abstract])) OR (Telecomm[Title/Abstract])) OR (Telecom[Title/Abstract]) | 25,376 |
|  | 1. (((((((((((Telerehabilitation[Title/Abstract]) OR (Telerehabilitations[Title/Abstract])) OR (Virtual Rehabilitation[Title/Abstract])) OR (Rehabilitations, Virtual[Title/Abstract])) OR (Rehabilitation, Virtual[Title/Abstract])) OR (Virtual Rehabilitations[Title/Abstract])) OR (Tele-rehabilitation[Title/Abstract])) OR (Tele rehabilitation[Title/Abstract])) OR (Remote Rehabilitation[Title/Abstract])) OR (Rehabilitation, Remote[Title/Abstract])) OR (Rehabilitations, Remote[Title/Abstract])) OR (Remote Rehabilitations[Title/Abstract]) | 13,601 |
|  | 1. (((((((((Teletherapy[Title/Abstract]) OR (Teletherapy, Mental Health[Title/Abstract])) OR (Telepsychotherapy[Title/Abstract])) OR (Telepsychology[Title/Abstract])) OR (Counseling, Distance[Title/Abstract])) OR (E-Counseling[Title/Abstract])) OR (E Counseling[Title/Abstract])) OR (E-Therapy[Title/Abstract])) OR (E-Therapies[Title/Abstract])) OR (E Therapy[Title/Abstract]) | 3,949 |
|  | 1. (((#1) OR (#2)) OR (#3)) OR (#4) | 82,976 |
| Reimbursement | 1. ((((((((((((((((((((((((((((((((((((((((Reimbursement[Title/Abstract]) OR (Incentive Reimbursements[Title/Abstract])) OR (Incentive Reimbursement[Title/Abstract])) OR (Pay for Performance[Title/Abstract])) OR (Performance, Pay for[Title/Abstract])) OR (Disproportionate Reimbursement[Title/Abstract])) OR (Disproportionate Reimbursements[Title/Abstract])) OR (Reimbursement, Disproportionate[Title/Abstract])) OR (Reimbursements, Disproportionate[Title/Abstract])) OR (Disproportionate Share Reimbursement[Title/Abstract])) OR (Disproportionate Share Reimbursements[Title/Abstract])) OR (Reimbursements, Disproportionate Share[Title/Abstract])) OR (Share Reimbursement, Disproportionate[Title/Abstract])) OR (Share Reimbursements, Disproportionate[Title/Abstract])) OR (Mechanism, Reimbursement[Title/Abstract])) OR (Mechanisms, Reimbursement[Title/Abstract])) OR (Reimbursement Mechanism[Title/Abstract])) OR (Reimbursement, Health Insurance[Title/Abstract])) OR (Third-Party Payments[Title/Abstract])) OR (Payment, Third-Party[Title/Abstract])) OR (Payments, Third-Party[Title/Abstract])) OR (Third Party Payments[Title/Abstract])) OR (Third-Party Payment[Title/Abstract])) OR (Health Insurance Reimbursement[Title/Abstract])) OR (Health Insurance Reimbursements[Title/Abstract])) OR (Insurance Reimbursement, Health[Title/Abstract])) OR (Insurance Reimbursements, Health[Title/Abstract])) OR (Reimbursements, Health Insurance[Title/Abstract])) OR (Third-Party Payers[Title/Abstract])) OR (Payer, Third-Party[Title/Abstract])) OR (Payers, Third-Party[Title/Abstract])) OR (Third Party Payers[Title/Abstract])) OR (Third-Party Payer[Title/Abstract])) OR (Reimbursement, Prospective[Title/Abstract])) OR (Prospective Reimbursement[Title/Abstract])) OR (Prospective Reimbursements[Title/Abstract])) OR (Reimbursements, Prospective[Title/Abstract])) OR (Fee-for-Service Reimbursement[Title/Abstract])) OR (Fee-for-Service Reimbursements[Title/Abstract])) OR (Reimbursement, Fee for Service[Title/Abstract])) OR (Reimbursements, Fee-for-Service[Title/Abstract]) | 37,258 |
|  | 1. ((((((((((Pay[Title/Abstract]) OR (Payments[Title/Abstract])) OR (Payment[Title/Abstract])) OR (Refund[Title/Abstract])) OR (Repay[Title/Abstract])) OR (Repayments[Title/Abstract])) OR (Repayment[Title/Abstract])) OR (Capitation[Title/Abstract])) OR (Bundled payments[Title/Abstract])) OR (Value-based care[Title/Abstract])) OR (Diagnosis-based payments[Title/Abstract]) | 92,045 |
|  | 1. (#6) OR (#7) | 1,20,221 |
| Final search | 1. (#5) AND (#8) | 1,972 |

**Search strategy: Scopus**

| Keyword | Search syntax | No. of hits |
| --- | --- | --- |
| Telemedicine | 1. (TITLE-ABS-KEY (telemedicine OR "virtual W/1 medicine" OR "tele referral" OR "health W/1 mobile" OR mhealth OR telehealth OR ehealth OR "tele intensive care” OR "tele icu" OR "tele care")) | 116,134 |
|  | 1. (TITLE-ABS-KEY (“virtual W/1 rehabilitation*”) OR "tele rehabilitation" OR (“remote W/1 rehabilitation*”)) | 3,417 |
|  | 1. (TITLE-ABS-KEY (teletherapy OR "teletherapy mental health" OR (“teletherapy W/1 mental-health”) telepsychotherap* OR telepsychology OR (counsel* W/1 distance) OR "e counsel*" OR "e therap*")) | 57 |
|  | 1. (TITLE-ABS-KEY (telemonitor OR "remote monitoring" OR teleconsultation* OR (consultation W/1 remote) OR telediagnosis OR telepsychiatry OR "telecom" OR "telecomm")) | 64,951 |
|  | 1. #1 OR #2 OR #3 OR #4 | 172,327 |
| Reimbursement | 1. (TITLE-ABS-KEY (reimbursement* OR "incentive reimbursement*" OR "pay for performance" OR ("disproportionate W/1 reimbursement*") OR "disproportionate share reimbursement*" OR (“disproportionate-share W/1 reimbursement”) OR ("reimbursement W/1 mechanism*") OR "health-insurance W/1 reimbursement*" OR "third-party W/1 pay*" OR "prospective W/1 reimbursement*" OR "fee-for-service W/1 reimbursement*")) | 80,381 |
|  | 1. (TITLE-ABS-KEY (pay OR payment* OR refund OR repay OR repayment* OR capitation OR "bundled payment*" OR "value based care" OR "diagnosis-based payments")) | 379,993 |
|  | 1. #6 OR #7 | 442,260 |
| Final search | 1. #5 AND #8 | 4,251 |

**Search strategy: Web of Science**

| Keyword | Search Syntax | No. of hits |
| --- | --- | --- |
| Telemedicine | 1. ALL=(Telemedicine OR Tele-Referral OR “Tele Referral” OR “Tele-Referrals” OR “Virtual Medicine” OR Medicine OR Virtual OR eHealth OR Telecare OR Tele-Care OR “Tele Care” OR “Tele-Intensive Care” OR “Tele Intensive Care” OR “Tele-ICU” OR “Tele ICU” OR “Mobile Health” OR Health OR Mobile OR mHealth ) | 24,656,586 |
|  | 1. ALL=(Teleconsultation OR Teleconsultations OR Consultation OR Remote ) | 754,171 |
| Reimbursement | 1. (ALL=(Reimbursement OR “Incentive Reimbursements” OR “Incentive Reimbursement” OR “Pay for Performance” OR “Performance Pay for” OR “Disproportionate Reimbursement” OR “Disproportionate Reimbursements” OR “Reimbursement Disproportionate” OR “Reimbursements Disproportionate” OR “Disproportionate Share Reimbursement” OR “Disproportionate Share Reimbursements” OR “Reimbursements Disproportionate Share” OR “Share Reimbursement” OR “Disproportionate Share Reimbursements” OR “Disproportionate Mechanism Reimbursement” OR “Mechanisms Reimbursement” OR “Reimbursement Mechanism” OR “Reimbursement Health Insurance” OR “Third-Party Payments” OR “Payment Third-Party” OR “Third Party Payments” )) OR ALL=(“Third-Party Payment” OR “Health Insurance Reimbursement” OR “Health Insurance Reimbursements” OR “Insurance Reimbursement” OR “Health Insurance Reimbursements” OR “Health Reimbursements” OR “Health Insurance” OR “Third-Party Payers” OR “Payer Third-Party” OR “Payers Third-Party” OR “Third Party Payers” OR “Third-Party Payer” OR “Reimbursement Prospective” OR “Prospective Reimbursement” OR “Prospective Reimbursements” OR “Reimbursements Prospective” OR “Fee-for-Service Reimbursement” OR “Fee-for-Service Reimbursements” OR “Reimbursement Fee for Service” OR “Reimbursements Fee-for-Service”) | 97,234 |
|  | 1. #1 AND #2 AND #3 | 1,951 |

**Search strategy: Embase**

| **Keyword** | **Search Syntax** | **No. of hits** |
| --- | --- | --- |
| Telemedicine | 1. (Telemedicine or Tele-Referral or “Tele Referral” or “Tele-Referrals” or “Virtual Medicine” or Medicine or Virtual or eHealth or Telecare or Tele-Care or “Tele Care” or “Tele-Intensive Care” or “Tele Intensive Care” or “Tele-ICU” or “Tele ICU” or “Mobile Health” or Health or Mobile or mHealth).mp. [mp=title, abstract, heading word, drug trade name, original title, device manufacturer, drug manufacturer, device trade name, keyword heading word, floating subheading word, candidate term word] | 6,925,394 |
|  | 1. (Teleconsultation or Teleconsultations or Consultation or Remote).mp. [mp=title, abstract, heading word, drug trade name, original title, device manufacturer, drug manufacturer, device trade name, keyword heading word, floating subheading word, candidate term word] | 365,680 |
| Reimbursement | 1. (Reimbursement or “Incentive Reimbursements” or “Incentive Reimbursement” or “Pay for Performance” or “Performance Pay for” or “Disproportionate Reimbursement” or “Disproportionate Reimbursements” or “Reimbursement Disproportionate” or “Reimbursements Disproportionate” or “Disproportionate Share Reimbursement” or “Disproportionate Share Reimbursements” or “Reimbursements Disproportionate Share” or “Share Reimbursement” or “Disproportionate Share Reimbursements” or “Disproportionate Mechanism Reimbursement” or “Mechanisms Reimbursement” or “Reimbursement Mechanism” or “Reimbursement Health Insurance” or “Third-Party Payments” or “Payment Third-Party” or “Third Party Payments” or “Third-Party Payment” or “Health Insurance Reimbursement” or “Health Insurance Reimbursements” or “Insurance Reimbursement” or “Health Insurance Reimbursements” or “Health Reimbursements” or “Health Insurance” or “Third-Party Payers” or “Payer Third-Party” or “Payers Third-Party” or “Third Party Payers” or “Third-Party Payer” or “Reimbursement Prospective” or “Prospective Reimbursement” or “Prospective Reimbursements” or “Reimbursements Prospective” or “Fee-for-Service Reimbursement” or “Fee-for-Service Reimbursements” or “Reimbursement Fee for Service” or “Reimbursements Fee-for-Service”).mp. [mp=title, abstract, heading word, drug trade name, original title, device manufacturer, drug manufacturer, device trade name, keyword heading word, floating subheading word, candidate term word] | 277,624 |
|  | 1. 1 and 2 and 3 | 6,391 |
|  | 1. limit 4 to (human and nglish language and yr=”1990 -Current”) | 5,390 |

**Search strategy: Global Health**

| **Keyword** | **Search Syntax** | **No. of hits** |
| --- | --- | --- |
| Telemedicine | 1. (telemedicine or "virtual medicine" or "tele-referral*" or "mobile health" or "health adj1 mobile" or mhealth or telehealth or ehealth or "tele-intensive-care" or "tele-icu" or "tele-care").mp. [mp=abstract, title, original title, heading words, cabicodes words] | 7,838 |
|  | 1. ("tele-rehabilitation*" or "virtual adj1 rehabilitation*" or "virtual-rehabilitation" or "remote adj1 rehabilitation*" or "remote-rehabilitation").mp. [mp=abstract, title, original title, heading words, cabicodes words] | 30 |
|  | 1. (teletherapy or "teletherapy adj1 mental-health" or telepsychotherap* or telepsychology or (counsel* adj1 distance) or "distance-counsel*" or "e-counsel*" or "e-therap*").mp. [mp=abstract, title, original title, heading words, cabicodes words] | 174 |
|  | 1. (telemonitor or "remote-monitor*" or teleconsultation* or (consultation adj1 remote) or "remote-consult*" or telediagnosis or telepsychiatry or "telecom*").mp. [mp=abstract, title, original title, heading words, cabicodes words] | 2,777 |
|  | 1. 1 or 2 or 3 or 4 | 9,629 |
| Reimbursement | 1. (reimbursement* or "incentive reimbursement*" or "pay-for adj1 performance" or "disproportionate adj1 reimbursement*" or "disproportionate adj2 share reimbursement*" or "disproportionate-share-reimbursement" or "reimbursement adj1 mechanism*" or "health insurance adj2 reimbursement*" or "third party adj2 pay*" or "prospective adj1 reimbursement*" or "fee-for-service adj1 reimbursement*").mp. [mp=abstract, title, original title, heading words, cabicodes words] | 3.446 |
|  | 1. (pay or payment* or refund or repay or repayment* or capitation or "bundled-payment*" or "value-based-care" or "diagnosis-based payments").mp. [mp=heading words, abstract, title, country as subject] | 22,809 |
|  | 1. 6 or 7 | 25,665 |
|  | 1. 5 and 8 | 259 |

**Search Strategy: ECONLIT**

| **Keyword** | **Search Syntax** | **No. of hits** |
| --- | --- | --- |
| Telemedicine | 1. (telemedicine or "virtual medicine" or "tele-referral*" or "mobile health" or "health adj1 mobile" or mhealth or telehealth or ehealth or "tele-intensive-care" or "tele-icu" or "tele-care").mp. [mp=heading words, abstract, title, country as subject] | 167 |
|  | 1. ("tele-rehabilitation*" or "virtual adj1 rehabilitation*" or "virtual-rehabilitation" or "remote adj1 rehabilitation*" or "remote-rehabilitation").mp. [mp=heading words, abstract, title, country as subject] | 1 |
|  | 1. (teletherapy or "teletherapy adj1 mental-health" or telepsychotherap* or telepsychology or (counsel* adj1 distance) or "distance-counsel*" or "e-counsel*" or "e-therap*").mp. [mp=heading words, abstract, title, country as subject] | 0 |
|  | 1. (telemonitor or "remote-monitor*" or teleconsultation* or (consultation adj1 remote) or "remote-consult*" or telediagnosis or telepsychiatry or "telecom*").mp. [mp=heading words, abstract, title, country as subject] | 13,375 |
|  | 1. 1 or 2 or 3 or 4 | 13,526 |
| Reimbursement | 1. (reimbursement* or "incentive reimbursement*" or "pay-for adj1 performance" or "disproportionate adj1 reimbursement*" or "disproportionate adj2 share reimbursement*" or "disproportionate-share-reimbursement" or "reimbursement adj1 mechanism*" or "health insurance adj2 reimbursement*" or "third party adj2 pay*" or "prospective adj1 reimbursement*" or "fee-for-service adj1 reimbursement*").mp. [mp=heading words, abstract, title, country as subject] | 1,414 |
|  | 1. (pay or payment* or refund or repay or repayment* or capitation or "bundled-payment*" or "value-based-care" or "diagnosis-based payments").mp. [mp=heading words, abstract, title, country as subject] | 76,666 |
|  | 1. 6 or 7 | 77,667 |
|  | 1. 5 and 8 | 437 |
