# Supplementary material for: Telemedicine Public Reimbursement Models for National and Subnational Jurisdictions: Scoping Review
Source: J Med Internet Res. 2025 Aug 12;27:e75478. doi: 10.2196/75478 (PMC12341443; doi:10.2196/75478)
Supplement: Multimedia Appendix 3 [file jmir-v27-e75478-s003.docx]

| **Country (Code)** | **Affiliation of interviewees / involvement in telemedicine** | **Responded via** |
| --- | --- | --- |
| Canada (KII Canada) | A manager of a healthcare facility that utilized telemedicine service | Email |
| India and the United States (KII India #1) | A physician-scientist specializing in digital health, artificial intelligence (AI), and governance and regional advisor for digital health | Interview |
| India (KII India #2) | Affiliated to India’s national telemedicine service - eSanjeevani | Interview |
| Nepal (KII Nepal) | A healthcare provider at Kathmandu Model Hospital | Interview |
| Taiwan (KII Taiwan) | An HTA Researcher at Center for Drug Evaluation, Taiwan/ | Interview and email |
