# Supplementary material for: Telemedicine Public Reimbursement Models for National and Subnational Jurisdictions: Scoping Review
Source: J Med Internet Res. 2025 Aug 12;27:e75478. doi: 10.2196/75478 (PMC12341443; doi:10.2196/75478)
Supplement: Multimedia Appendix 4 [file jmir-v27-e75478-s004.docx]

| Reimbursement Considerations | Sources by jurisdictions mentioning the criteria | | | | | | |
| --- | --- | --- | --- | --- | --- | --- | --- |
|  | US | | Canada | | Europe | | Asia and Australia |
| **Purpose** | | | | | | | |
| Prevention/promotion | [1, 2] | |  | |  | |  |
| Diagnosis, treatment, follow-up, patient support, and management | [1-17];  KII India #1 | | [18];  KII Canada | | [19-22] | | [23];  KII India #1, KII India #2, KII Taiwan, KII Nepal |
| Administrative services | [10] | |  | |  | |  |
| **Health condition** | | | | | | | |
| Physical health | [1-3, 6, 10-13, 15-17, 24];  KII India #1 | | [5];  KII Canada | | [21] | | KII India #1, KII India #2, KII Taiwan, KII Nepal |
| Mental health | [3, 15, 16, 24] | | [18];  KII Canada | | [5] | | KII Taiwan |
| **Patient’s non-health condition** | | | | | | | |
| Patient remoteness | [13, 16, 25] | | KII Canada | |  | | KII India #1, KII India #2 |
| The patient has established a relationship with a provider^b^ | [16] | |  | |  | |  |
| Patient vulnerability | [26] | |  | | [21] | |  |
| Service-related^c^ | [16, 24] | |  | |  | |  |
| Urban residence | [16] | | KII Canada | |  | | KII India #1, KII India #2 |
| **Service provider** | | | | | | | |
| Physicians | [1, 4, 6-10, 16, 17, 24];  KII India #1 | | [18];  KII Canada | | [19-22] | | KII India #1, KII India #2, KII Taiwan, KII Nepal |
| Nurses | [2, 4, 10, 12, 16, 17, 27, 28];  KII India #1 | | [5];  KII Canada | | [21] | | KII India #1, KII India #2, KII Taiwan, KII Nepal |
| Pharmacist | [1, 11, 14] |  | | [21] | |  | |
| Social worker | [4, 16] |  | | [21] | |  | |
| Other ^a^ | [1, 2, 4, 14, 16] |  | | [19, 22] | |  | |
| **Interaction** | | | | | | | |
| Provider to patients | [2-4, 6-9, 13-17, 19, 25, 26, 28-30];  KII India #1 | | [18];  KII Canada | | [21, 22, 31] | | [23];  KII India #1, KII India #2, KII Taiwan, KII Nepal |
| Provider to provider | [10, 12, 16, 24];  KII India #1 | | [18] | |  | | KII India #1, KII India #2, KII Taiwan, KII Nepal |
| **Time** | | | | | | | |
| Interactive | [1, 2, 4, 6, 7, 9, 10, 12, 14, 16, 25, 26, 28] | | [18] | | [22, 31] | | [23];  KII India #1, KII India #2, KII Taiwan, KII Nepal |
| Store and forward | [1, 13, 16, 26];  KII India #1 | | [18] | |  | | KII Taiwan, KII Nepal |
| Remote monitoring | [1, 7, 15-17] | |  | | [22] | |  |
| **Technology** | | | | | | | |
| Video-based | [1, 7, 9, 10, 12, 13, 16, 17, 27, 29, 30];  KII India #1 | | [18] | | [22, 31] | | [23], KII India #1, KII India #2 |
| Audio-based | [16];  KII India #1 | | [18] | | [22, 31] | | [23];  KII India #1, KII India #2, KII Nepal, KII Taiwan |
| Software applications | [8] | |  | | [22, 31] | | KII India #1, KII India #2, KII Nepal, KII Taiwan |
| Messaging services | [2, 17] | |  | | [31] | |  |
| Physical devices |  | |  | | [22] | |  |
| Website | [16] | | KII Canada | |  | | KII India #1, KII India #2 |

KII: key informant interview

**References**

1. Ranganathan, C. and S. Balaji. Key Factors Affecting the Adoption of Telemedicine by Ambulatory Clinics: Insights from a Statewide Survey*.* Telemed J E Health. 2020; 26(2): p. 218-225. [doi: 10.1089/tmj.2018.0114]

2. Wharton, M.K., et al. Qualitative Analysis of Health Systems Utilizing Non-Face-to-Face Chronic Care Management for Medicare-Insured Patients With Diabetes*.* J Ambul Care Manage. 2020; 43(4): p. 326-334. [doi: 10.1097/jac.0000000000000342]

3. Zhao, M., et al. Telehealth: Advances in Alternative Payment Models*.* Telemed J E Health. 2020; 26(12): p. 1492-1499. [doi: 10.1089/tmj.2019.0294]

4. Ward, M.M., et al. Comparison of Telehealth and In-Person Behavioral Health Services and Payment in a Large Rural Multisite Usual Care Study*.* Telemed J E Health. 2023; 29(11): p. 1613-1623. [doi: 10.1089/tmj.2022.0445]

5. Wang, B., et al. Association Between Telemedicine Use in Nonmetropolitan Counties and Quality of Care Received by Medicare Beneficiaries With Serious Mental Illness*.* JAMA Netw Open. 2022; 5(6): p. e2218730. [doi: 10.1001/jamanetworkopen.2022.18730]

6. Salko, L., D. Terry, and D. Weingard. The Path To Value Based Care : Benefits For Patients, Endocrinologists, And The Healthcare System (Includes Case Studies). Proceedings of AACE Annual Meeting; 2024. New Orleans, United States. AACE; 2024.

7. Rogove, H.J. and J. Bernard. Reimbursement and Payment Models for Tele-ICU. in M.A. Koenig. Telemedicine in the ICU. Cham: Springer International Publishing; 2019. p. 45-61.

8. Rajan, B., et al. The Promise of mHealth for Chronic Disease Management Under Different Payment Systems*.* Manufacturing & Service Operations Management. 2022; 24. [doi: 10.1287/msom.2022.1143]

9. Myers, K.M., J.M. Valentine, and S.M. Melzer. Feasibility, acceptability, and sustainability of telepsychiatry for children and adolescents*.* Psychiatr Serv. 2007; 58(11): p. 1493-6. [doi: 10.1176/ps.2007.58.11.1493]

10. Mohr, N.M., et al. Provider-to-provider telehealth for sepsis patients in a cohort of rural emergency departments*.* Acad Emerg Med. 2024; 31(4): p. 326-338. [doi: 10.1111/acem.14857]

11. Martin, R., et al. Financial performance and reimbursement of pharmacist-led chronic care management*.* American Journal of Health-System Pharmacy. 2020; 77(23): p. 1973-1979. [doi: 10.1093/ajhp/zxaa300]

12. Komet, H. An Analysis of the Home Health Marketplace: How Telehealth Technology May Assist Home Health Agencies with Changes in Home Care Delivery under the Prospective Payment System*.* Home Health Care Management & Practice. 2001; 13(2): p. 142-148. [doi: 10.1177/108482230101300209]

13. Federal Register. Medicare program; revisions to payment policies under the physician fee schedule, and other Part B payment policies for CY 2008; revisions to the payment policies of ambulance services under the ambulance fee schedule for CY 2008; and the amendment of the e-prescribing exemption for computer generated facsimile transmissions. Final rule with comment period. 2007.

14. Cohen, G., et al. How CPC+ supported patient care during the COVID-19 pandemic: Lessons for alternative payment models*.* Healthc (Amst). 2024; 12(2): p. 100745. [doi: 10.1016/j.hjdsi.2024.100745]

15. Chen, J., T.K. Maguire, and M. Qi Wang. Telehealth Infrastructure, Accountable Care Organization, and Medicare Payment for Patients with Alzheimer's Disease and Related Dementia Living in Socially Vulnerable Areas*.* Telemed J E Health. 2024; 30(8): p. 2148-2156. [doi: 10.1089/tmj.2024.0119]

16. Bernard, J. and M.W. Kwong. Federal and State Policies on Telehealth Reimbursement. in R. Latifi, C.R. Doarn, and R.C. Merrell. Telemedicine, Telehealth and Telepresence: Principles, Strategies, Applications, and New Directions. Cham: Springer International Publishing; 2021. p. 115-127.

17. Bauer, G. Delivering Value-Based Care With E-Health Services*.* J Healthc Manag. 2018; 63(4): p. 251-260. [doi: 10.1097/jhm-d-18-00077]

18. Fu, R., et al. Virtual and in-person visits by Ontario physicians in the COVID-19 era*.* J Telemed Telecare. 2022; 30(4): p. 706-714. [doi: 10.1177/1357633x221086447]

19. Eddison, N., et al. Telehealth provision across allied health professions (AHP): An investigation of reimbursement considerations for its successful implementation in England*.* Health Sci Rep. 2023; 6(1): p. e991. [doi: 10.1002/hsr2.991]

20. Grandchamp, C. and L. Gardiol. Does a mandatory telemedicine call prior to visiting a physician reduce costs or simply attract good risks? Health Econ. 2011; 20(10): p. 1257-67. [doi: 10.1002/hec.1668]

21. Lluch, M. Incentives for telehealthcare deployment that support integrated care: a comparative analysis across eight European countries*.* Int J Integr Care. 2013; 13: p. e042. [doi: 10.5334/ijic.1062]

22. Raes, S., et al. Physicians’ views on optimal use and payment system for telemedicine: a qualitative study*.* BMC Health Services Research. 2023; 23(1): p. 292. [doi: 10.1186/s12913-023-09314-w]

23. Ryan, M., et al. Changing from telephone to videoconference for pre-treatment pharmacist consults in cancer services: Impacts to funding and time efficiency*.* J Telemed Telecare. 2021; 27(10): p. 680-684. [doi: 10.1177/1357633x211048393]

24. Guterman, E.L., et al. Care Ecosystem Collaborative Model and Health Care Costs in Medicare Beneficiaries With Dementia: A Secondary Analysis of a Randomized Clinical Trial*.* JAMA Internal Medicine. 2023; 183(11): p. 1222-1228. [doi: 10.1001/jamainternmed.2023.4764]

25. Johnson, K.A., et al. Using telemedicine interventions during COVID-19 to expand care post COVID-19*.* Am J Manag Care. 2023; 29(1): p. e31-e35. [doi: 10.37765/ajmc.2023.89311]

26. Timbie, J.W., et al. Impact of global payments for uninsured uncompensated care: evaluating california’s global payment program (GPP). Proceedings of Abstracts from the 2020 Annual Meeting of the Society of General Internal Medicine; 2020. J Gen Intern Med; 2020.

27. Rambur, B., M.V. Palumbo, and M. Nurkanovic. Prevalence of Telehealth in Nursing: Implications for Regulation and Education in the Era of Value-Based Care*.* Policy Polit Nurs Pract. 2019; 20(2): p. 64-73. [doi: 10.1177/1527154419836752]

28. Rumberger, J.S. and K. Dansky. Is there a business case for telehalth in home health agencies? Telemed J E Health. 2006; 12(2): p. 122-27. [doi: <https://doi.org/10.1089/tmj.2006.12.122>]

29. Powers, B.W., et al. Association Between Primary Care Payment Model and Telemedicine Use for Medicare Advantage Enrollees During the COVID-19 Pandemic*.* JAMA Health Forum. 2021; 2(7): p. e211597-e211597. [doi: 10.1001/jamahealthforum.2021.1597]

30. Filippi, M.K., et al. COVID-19's Financial Impact on Primary Care Clinicians and Practices*.* J Am Board Fam Med. 2021; 34(3): p. 489-497. [doi: 10.3122/jabfm.2021.03.200502]

31. Dahlgren, C., et al. Short- and intermediate-term impact of DTC telemedicine consultations on subsequent healthcare consumption*.* Eur J Health Econ. 2024; 25(1): p. 157-176. [doi: 10.1007/s10198-023-01572-z]
