# Supplementary material for: Telemedicine Public Reimbursement Models for National and Subnational Jurisdictions: Scoping Review
Source: J Med Internet Res. 2025 Aug 12;27:e75478. doi: 10.2196/75478 (PMC12341443; doi:10.2196/75478)
Supplement: Multimedia Appendix 5 [file jmir-v27-e75478-s005.docx]

| Models | Jurisdictions | Conditions | Pros/Cons |
| --- | --- | --- | --- |
| Prospective payment [1, 2] | - US [1, 2] | - Chronic conditions [2] | Not reported |
| Fee-for-service [3-21] and payment per contact [22] | - US [3, 4, 6-8, 10, 12-17, 19-21] - Canada [11] - Belgium [5] - Estonia [9] - Germany [9] - The Netherlands [9] - Switzerland [18] | - Chronic conditions [3, 4, 9, 15] - Dementia [14] - Mental health [11, 16] - COVID-19 [11] - Sepsis treated in emergency departments [8] | *Pros*   - Relatively preferred by service providers as it incentivizes service volume (KII Nepal, KII Taiwan)   *Cons*   - Fee-for-service reimbursement prioritizes volume over quality [3]; KII Taiwan] - May trigger overuse of services [22] |
| Capitation [4, 13] | US [4, 13] | - End stage renal disease related services [13] | *Pro*   - Allows for risk-adjustment according to the patient profile [4]   *Cons*   - Risk profile may not be directly observable [4] |
| Episode-based [23] | - US [23] | - Not reported | *Pro*   - Maximizes the number of patients [23] - May reduce the costs marginally, as providers are expected to manage the episode within the prospective payment reimbursement formula [23] |
| Case-based [9] and episode-based [23] | - Estonia [9] - Germany [9] - France [9] - Italy [9] - Spain [9] | - Inpatient services [9] | Not reported |
| Activity-based funding [9, 24] | - Australia [24] - Denmark [9] | - Cancer services [24] | Not reported |
| Blended model^a^ [9, 25] | - US [25] - Denmark [9] - Estonia [9] - Germany [9] - Spain [9] - The Netherlands [9] | - Primary care services [25] | Not reported |
| **Value-based models** | | | |
| Accountable Care Organization [6, 15, 21, 26, 27] | US [6, 15, 21, 26, 27] | - Stroke care, psychiatric and addiction [26] - Immunization and minor procedures [26] - Post-discharge monitoring [26] - Chronic care management [26] | *Pros*   - Hospitals were more likely to provide telehealth services compared to other payment models [26] - Prevent unnecessary hospitalizations and adoption of low-value technology [21] - Providers may face financial risk since they will be responsible for managing care within a fixed budget [21]   *Cons*   - May discourage initial adoption of new technologies due to cost concerns [26] |
| Quality-adjusted outcome capitation [6, 9] | - US [6] - Italy [9] - Spain [9] - UK [9] | - Primary care services [9] | *Pros*   - Offers the benefits of both outcome-based payment and capitation [9] |
| Bundled payments [9, 21, 26, 28, 29] | - US [9, 21, 26, 28, 29] - The Netherlands [9] | - Intensive and critical care [28] - Chronic condition [9] | Not reported |
| Unspecified value-based model^a^ [9, 30, 31] | - US [31] - UK [9] | - Elective care [9] - Emergency care [9] - Outpatients [9] - Diabetes and chronic care [31] | *Pros*   - Improves health outcomes [31] - Reduce healthcare resources [31] |

**References**

1. Timbie, J.W., et al. Impact of global payments for uninsured uncompensated care: evaluating california’s global payment program (GPP). Proceedings of Abstracts from the 2020 Annual Meeting of the Society of General Internal Medicine; 2020. J Gen Intern Med; 2020.

2. Komet, H. An Analysis of the Home Health Marketplace: How Telehealth Technology May Assist Home Health Agencies with Changes in Home Care Delivery under the Prospective Payment System*.* Home Health Care Management & Practice. 2001; 13(2): p. 142-148. [doi: 10.1177/108482230101300209]

3. Wharton, M.K., et al. Qualitative Analysis of Health Systems Utilizing Non-Face-to-Face Chronic Care Management for Medicare-Insured Patients With Diabetes*.* J Ambul Care Manage. 2020; 43(4): p. 326-334. [doi: 10.1097/jac.0000000000000342]

4. Rajan, B., et al. The Promise of mHealth for Chronic Disease Management Under Different Payment Systems*.* Manufacturing & Service Operations Management. 2022; 24. [doi: 10.1287/msom.2022.1143]

5. Raes, S., et al. Physicians’ views on optimal use and payment system for telemedicine: a qualitative study*.* BMC Health Services Research. 2023; 23(1): p. 292. [doi: 10.1186/s12913-023-09314-w]

6. Powers, B.W., et al. Association Between Primary Care Payment Model and Telemedicine Use for Medicare Advantage Enrollees During the COVID-19 Pandemic*.* JAMA Health Forum. 2021; 2(7): p. e211597-e211597. [doi: 10.1001/jamahealthforum.2021.1597]

7. Myers, K.M., J.M. Valentine, and S.M. Melzer. Feasibility, acceptability, and sustainability of telepsychiatry for children and adolescents*.* Psychiatr Serv. 2007; 58(11): p. 1493-6. [doi: 10.1176/ps.2007.58.11.1493]

8. Mohr, N.M., et al. Provider-to-provider telehealth for sepsis patients in a cohort of rural emergency departments*.* Acad Emerg Med. 2024; 31(4): p. 326-338. [doi: 10.1111/acem.14857]

9. Lluch, M. Incentives for telehealthcare deployment that support integrated care: a comparative analysis across eight European countries*.* Int J Integr Care. 2013; 13: p. e042. [doi: 10.5334/ijic.1062]

10. Johnson, K.A., et al. Using telemedicine interventions during COVID-19 to expand care post COVID-19*.* Am J Manag Care. 2023; 29(1): p. e31-e35. [doi: 10.37765/ajmc.2023.89311]

11. Fu, R., et al. Virtual and in-person visits by Ontario physicians in the COVID-19 era*.* J Telemed Telecare. 2022; 30(4): p. 706-714. [doi: 10.1177/1357633x221086447]

12. Filippi, M.K., et al. COVID-19's Financial Impact on Primary Care Clinicians and Practices*.* J Am Board Fam Med. 2021; 34(3): p. 489-497. [doi: 10.3122/jabfm.2021.03.200502]

13. Federal Register. Medicare program; revisions to payment policies under the physician fee schedule, and other Part B payment policies for CY 2008; revisions to the payment policies of ambulance services under the ambulance fee schedule for CY 2008; and the amendment of the e-prescribing exemption for computer generated facsimile transmissions. Final rule with comment period. 2007.

14. Chen, J., T.K. Maguire, and M. Qi Wang. Telehealth Infrastructure, Accountable Care Organization, and Medicare Payment for Patients with Alzheimer's Disease and Related Dementia Living in Socially Vulnerable Areas*.* Telemed J E Health. 2024; 30(8): p. 2148-2156. [doi: 10.1089/tmj.2024.0119]

15. Bauer, G. Delivering Value-Based Care With E-Health Services*.* J Healthc Manag. 2018; 63(4): p. 251-260. [doi: 10.1097/jhm-d-18-00077]

16. Ward, M.M., et al. Comparison of Telehealth and In-Person Behavioral Health Services and Payment in a Large Rural Multisite Usual Care Study*.* Telemed J E Health. 2023; 29(11): p. 1613-1623. [doi: 10.1089/tmj.2022.0445]

17. Guterman, E.L., et al. Care Ecosystem Collaborative Model and Health Care Costs in Medicare Beneficiaries With Dementia: A Secondary Analysis of a Randomized Clinical Trial*.* JAMA Internal Medicine. 2023; 183(11): p. 1222-1228. [doi: 10.1001/jamainternmed.2023.4764]

18. Grandchamp, C. and L. Gardiol. Does a mandatory telemedicine call prior to visiting a physician reduce costs or simply attract good risks? Health Econ. 2011; 20(10): p. 1257-67. [doi: 10.1002/hec.1668]

19. Wang, B., et al. Association Between Telemedicine Use in Nonmetropolitan Counties and Quality of Care Received by Medicare Beneficiaries With Serious Mental Illness*.* JAMA Netw Open. 2022; 5(6): p. e2218730. [doi: 10.1001/jamanetworkopen.2022.18730]

20. Martin, R., et al. Financial performance and reimbursement of pharmacist-led chronic care management*.* American Journal of Health-System Pharmacy. 2020; 77(23): p. 1973-1979. [doi: 10.1093/ajhp/zxaa300]

21. Rambur, B., M.V. Palumbo, and M. Nurkanovic. Prevalence of Telehealth in Nursing: Implications for Regulation and Education in the Era of Value-Based Care*.* Policy Polit Nurs Pract. 2019; 20(2): p. 64-73. [doi: 10.1177/1527154419836752]

22. Dahlgren, C., et al. Short- and intermediate-term impact of DTC telemedicine consultations on subsequent healthcare consumption*.* Eur J Health Econ. 2024; 25(1): p. 157-176. [doi: 10.1007/s10198-023-01572-z]

23. Rumberger, J.S. and K. Dansky. Is there a business case for telehalth in home health agencies? Telemed J E Health. 2006; 12(2): p. 122-27. [doi: <https://doi.org/10.1089/tmj.2006.12.122>]

24. Ryan, M., et al. Changing from telephone to videoconference for pre-treatment pharmacist consults in cancer services: Impacts to funding and time efficiency*.* J Telemed Telecare. 2021; 27(10): p. 680-684. [doi: 10.1177/1357633x211048393]

25. Cohen, G., et al. How CPC+ supported patient care during the COVID-19 pandemic: Lessons for alternative payment models*.* Healthc (Amst). 2024; 12(2): p. 100745. [doi: 10.1016/j.hjdsi.2024.100745]

26. Zhao, M., et al. Telehealth: Advances in Alternative Payment Models*.* Telemed J E Health. 2020; 26(12): p. 1492-1499. [doi: 10.1089/tmj.2019.0294]

27. Ranganathan, C. and S. Balaji. Key Factors Affecting the Adoption of Telemedicine by Ambulatory Clinics: Insights from a Statewide Survey*.* Telemed J E Health. 2020; 26(2): p. 218-225. [doi: 10.1089/tmj.2018.0114]

28. Rogove, H.J. and J. Bernard. Reimbursement and Payment Models for Tele-ICU. in M.A. Koenig. Telemedicine in the ICU. Cham: Springer International Publishing; 2019. p. 45-61.

29. Bernard, J. and M.W. Kwong. Federal and State Policies on Telehealth Reimbursement. in R. Latifi, C.R. Doarn, and R.C. Merrell. Telemedicine, Telehealth and Telepresence: Principles, Strategies, Applications, and New Directions. Cham: Springer International Publishing; 2021. p. 115-127.

30. Eddison, N., et al. Telehealth provision across allied health professions (AHP): An investigation of reimbursement considerations for its successful implementation in England*.* Health Sci Rep. 2023; 6(1): p. e991. [doi: 10.1002/hsr2.991]

31. Salko, L., D. Terry, and D. Weingard. The Path To Value Based Care : Benefits For Patients, Endocrinologists, And The Healthcare System (Includes Case Studies). Proceedings of AACE Annual Meeting; 2024. New Orleans, United States. AACE; 2024.
